# Laparoscopic pancreaticoduodenectomy for metastatic pancreatic melanoma: A case report

**DOI:** 10.1097/MD.0000000000012940

**Published:** 2018-11-02

**Authors:** Xueqing Liu, Feng Feng, Tianyang Wang, Jianzhang Qin, Xiangyan Yin, Guiqing Meng, Changqing Yan, Zhongqiang Xing, Jiayue Duan, Chen Liu, Jianhua Liu

**Affiliations:** a Second Hospital of Hebei Medical University, Shijiazhuang; b People's Hospital of Pingxiang, Xingtai, Hebei; c Fudan University Shanghai Cancer Center, Shanghai, China.

**Keywords:** melanoma, metastases, pancreas, resection

## Abstract

**Rationale:**

Pancreatic metastases from other malignant tumors are an uncommon clinical condition and account for approximately 2% of all pancreatic malignancies. The most common primary malignancy that metastasizes to pancreas is renal cell cancer. We reported a rare clinical case of metastatic melanoma to pancreas who underwent a successful laparoscopic pancreaticoduodenectomy (LPD) at our department.

**Patient concerns:**

A 54-year-old Chinese man complaining an unexplained jaundice was found to have a pancreatic mass and he was diagnosed with cutaneous melanoma (CM) 6 years ago.

**Diagnoses:**

Contrast-enhanced computed tomography (CECT) revealed a solid hypovascular mass measuring about 3.1 × 2.4 cm localized at the junction of pancreatic head and uncinate process, which compressed the lower common bile duct resulting in expansion of the upstream bile ducts.

**Interventions:**

We performed an LPD and regional lymphadenectomy on this patient.

**Outcomes:**

This patient was discharged home on postoperative day 19. Postoperative pathological results revealed a malignant melanoma with negative margins. Immunohistochemical (IHC) findings also suggested a malignant pancreatic tumor accompanied by necrosis and pigmentation, which confirmed the pathological diagnosis. Immunoreactivity was strongly positive for anti-S-100 protein (+++) and positive for anti-Vimentin (+). The cancer cells were negative for CEA, CK8/18, P53, Violin, CK19, SMA with Ki-67 over 40%. So this pancreatic mass was proved to be a metastatic pancreatic melanoma from the primary cutaneous lesion. After LPD, this patient was followed up by readmission to hospital every 2 month in the first half year. The serum bilirubin and tumor markers such as CA199 were normal. CECT and did not find any newly developed neoplasm at the pancreas or metastasis at other organs. At the last follow-up at 6 months after LPD, the patient's general condition was acceptable and the physical examination and imaging studies revealed no significant findings of melanoma.

**Lessons:**

Metastatic pancreatic tumors are often associated with well-defined margins, tumor necrosis, enhancement, and distant metastases without pancreatic duct dilatation and parenchymal atrophy. As the most common type of metastatic pancreatic tumor, renal cell cancers tend to have higher attenuation values than that of primary pancreatic cancer, while they had similar attenuation values on the portal phase. Primary pancreatic cancer was always associated with an elevated CA199, total bilirubin, and fasting plasma glucose levels. Surgical resection for metastases to pancreas should be aggressively considered in selected patients due to its unique value of providing palliation and a chance to cure. For patients with unresectable lesions, new therapeutic protocols should be recommended such as the combination of BRAF with MEK inhibitor and PD-1 blocker with or without ipilimumab.

## 1 Introduction

Pancreatic metastases from other malignant tumors are an uncommon clinical condition and account for approximately 2% of all pancreatic malignancies.^[1]^ According to a review article of 418 patients diagnosed with metastatic pancreatic diseases, the primary tumors were renal cell cancer (70.1%), melanoma (9.1%), colorectal cancer (8.6%), breast cancer (4.5%), sarcoma (4.3%), and lung cancer (3.4%).^[1]^ As for abdominal metastases from stage IV melanoma, a 2017 study of 1623 patients demonstrated that the secondary malignancies could occur in the liver (42.9%), gastrointestinal (GI) tract (20.7%), adrenal glands (8.5%), pancreas (2.3%), spleen (6.7%), and multiple sites (18.8%).^[2]^ Only a few articles have reported the surgical outcomes of pancreatic resection for metastases from other malignant tumors. And there are no generally accepted guidelines focusing on the systemic treatments for these patients. According to a literature review by Cosimo, in the last several decades, the number of pancreatectomy for metastatic malignancies is gradually increasing with acceptable morbidity and mortality rate.^[1]^ However, the efficacy of metastasectomy for metastatic pancreatic malignancy remains controversial due to the insufficiency of clinical cases. Previously, most patients with a metastatic pancreatic malignancy are usually not candidates for surgical treatment due to their widespread disease. The patient with metastases confined to the pancreatic parenchyma at the time of diagnosis is a rare clinical case, accounting for 5% of all pancreatic neoplasms.^[3]^ Nevertheless, pancreatic resection has the unique potential to cure the disease, and definite benefit of surgery for patient survival has been already observed in metastatic renal cell cancer to the pancreas.^[4]^ As for metastases from other primary cancers, we also believe the unique value attached to surgical treatment include not only providing palliation but a chance to cure and gain long-term survival. The indication for pancreatic resection is limited to patients with a good general condition, adequate disease control of primary malignancy and imaging studies indicating tumor resectability.^[5]^ Here, in this study, we reported a rare clinical case of metastatic melanoma to pancreas who underwent successful laparoscopic pancreaticoduodenectomy (LPD) at our department. The current related literature was also reviewed. This case report was approved by the ethics committee of the Second Hospital of Hebei Medical University, Shijiazhuang, China. Informed written consent was obtained from the patient for publication of this case report and accompanying images.

## 2 Case report

**Figure 1:**
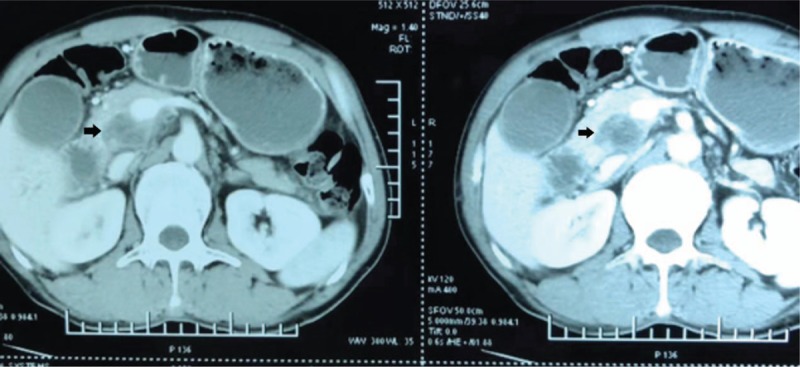
CECT showed a solid hypovascular mass measuring about 3.1 × 2.4 cm localized at the junction of pancreatic head and uncinate process (arrow head).

A pancreatic mass was observed in a 54-year-old Chinese man during a routine follow-up of cutaneous melanoma. Six years earlier, he had consulted a dermatologist with a progressively growing pigment mole after trauma on his back. After detailed imaging studies and other relevant examinations, he was diagnosed with malignant melanoma of stage T3N0M0, according to the 7th American Joint Committee on Cancer definition. This patient underwent extended surgical resection of the malignant lesion and immunotherapeutic treatments with IFNα-2b plus IL-2 on 1, 3, 6, 12, 18, 24, 30, 36 months after surgery (IFNα-2b, 3000,000 U, 15 times; IL-2, 1000,000 U, 15 times, intramuscular injection alternately). Postoperative pathological results also confirmed as malignant melanoma of stage T3N0M0. However, in year 4 following the index surgery, this patient complained of an upper abdominal discomfort but refused to receive further systemic examinations and treatments. In year 6, he presented with an unexplained jaundice of skin and was admitted to our department. The blood test showed a significantly elevated bilirubin level (total bilirubin, 153.4 μmol/L; direct bilirubin, 86.5 μmol/L) and a normal CA199 level of 33.3 U/mL (normal range < 40 U/mL). Contrast-enhanced computed tomography (CECT) revealed a solid hypovascular mass measuring about 3.1 × 2.4 cm localized at the junction of pancreatic head and uncinate process, which compressed the lower common bile duct resulting in expansion of the upstream bile ducts (Fig. 1). Percutaneous transhepatic catheter drainage was performed in this patient to reduce the serum concentration of serum bilirubin. Given the patient's acceptable general condition, good control of primary disease and imaging studies indicating tumor resectability, we obtained approval from him as well as his family and performed an LPD and regional lymphadenectomy on this patient. There were no complications following the surgery and the patient was discharged on day 19 after surgery.

**Figure 2:**
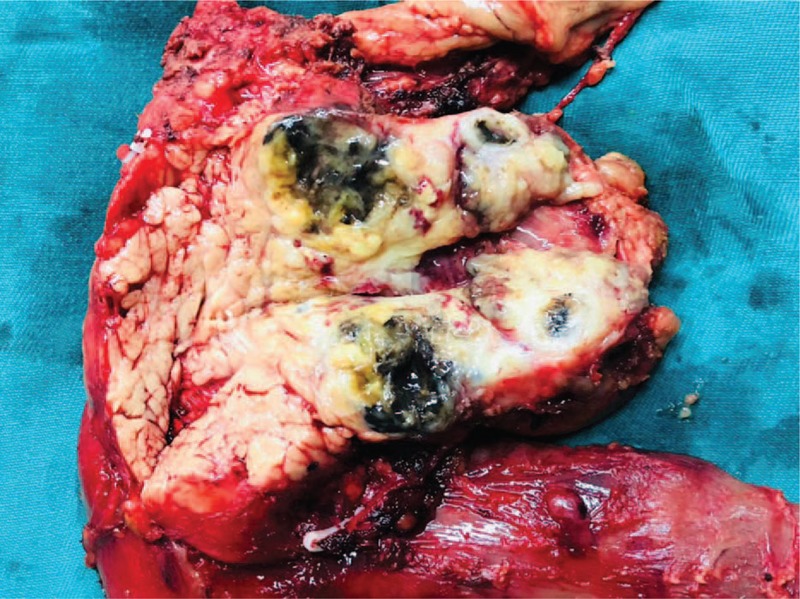
The macroscopic image of resected tumor specimen indicated a metastatic melanoma.

**Figure 3:**
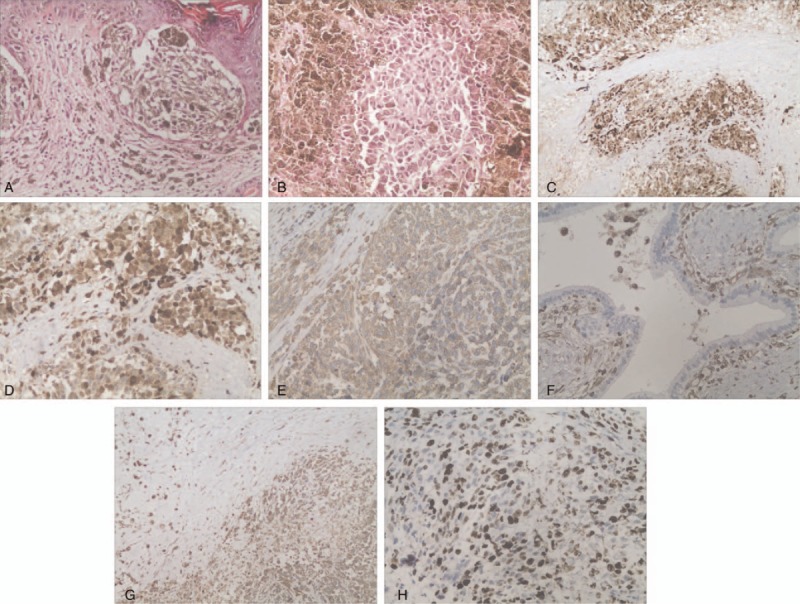
The IHC findings suggested a malignant pancreatic tumor accompanied by necrosis and pigmentation. (A) HE staining for the primary cutaneous melanoma (×200). (B) HE staining for the metastatic pancreatic melanoma (×200). (C1) Significantly positive IHC staining of S-100 protein (×200). (C2) Significantly positive IHC staining of S-100 (×400). (D1) Positive IHC staining of Vim (×200). (D2) Positive IHC staining of Vim (×400). (E1) IHC staining of Ki-67 over 40% (×100). (E2) IHC staining of Ki-67 over 40%(×200).

The gross morphology of the specimen is shown in Fig. 2. The pathological outcomes following the skin melanoma resection are given in Fig. 3A. Postoperative pathological results after LPD revealed a malignant melanoma with negative margins (Fig. 3B). Immunohistochemical (IHC) findings also suggested a malignant pancreatic tumor accompanied by necrosis and pigmentation, which confirmed the pathological diagnosis. Immunoreactivity was strongly positive for anti-S-100 protein (+++) and positive for anti-Vimentin (+) (Fig. C1-2 and D1-2). The cancer cells were negative for CEA, CK8/18, P53, Violin, CK19, SMA with Ki-67 over 40% (Fig. E1 and E2) So this pancreatic mass was confirmed to be a metastatic pancreatic melanoma from the primary cutaneous lesion.

After LPD, this patient was followed up by readmission to hospital every 2 months in the first half year. The serum bilirubin and tumor markers such as CA199 were normal. CECT and did not find any newly developed neoplasm at the pancreas or metastasis at other organs. At the last follow-up at 6 months after LPD, the patient's general condition was acceptable and the physical examination and imaging studies revealed no significant findings of melanoma.

## 3 Discussion

Although patients with pancreatic metastases are often complaining abdominal pain (47.5%), metastatic pancreatic tumors could also be asymptomatic in 32.5% of patients.^[6]^ They are more likely to be detected during regular follow-up after the index surgery for primary disease or as an unexpected finding on imaging study which is performed for an unrelated purpose.^[1]^

When patients with past histories of other malignancies develop pancreatic cancer, accurate preoperative diagnosis is essential for optimal treatment protocols which could affect the final choice of surgical or nonsurgical management. However, it is challenging to differentiate metastatic pancreatic malignancy from primary pancreatic cancer. To date, various methods have been developed and introduced to distinguish metastatic pancreatic malignancy from primary pancreatic cancer, such as CECT, positron emission tomography/computed tomography (PET/CT), and endoscopic ultrasound (EUS) with fine needle aspiration (FNA). Metastases are often considered when CECT imaging indicates a pancreatic mass with peripheral enhancement and low attenuation of the central area except for renal cell cancer. Lee et al noted that primary pancreatic cancer was more likely to show pancreatic duct dilatation and pancreatic atrophy, whereas metastatic pancreatic cancer tended to have well-defined margins, tumor necrosis, enhancement, and distant metastases.^[7]^ As the most common metastases to the pancreas, metastases from renal cell carcinoma should gain more attention than any other tumors, which are often mimicking a pancreatic neuroendocrine tumor. There is substantial overlap in radiological characteristics of these 2 entities. A recent study by Kim et al reported the relative percentage washout on CECT was helpful on this issue. In his initiative study, Kim et al found that the metastases from renal cell carcinoma were associated with considerably higher attenuation values than that of primary pancreatic cancer, while they had similar attenuation values on portal phase. This report also indicated that the metastases from renal cell carcinoma tended to show rapid wash out after the arterial period.^[8]^ The explanation for this phenomenon could be the metastases from renal cancer still inherit biological characteristics of high vascular perfusion from a primary tumor. Despite CECT, PET/CT has evolved as a novel diagnostic technique to differentiate pancreatic metastases and primary pancreatic cancer and detect unsuspected pancreatic metastases which cannot be detected by CECT. However, in a retrospective study by Hu, no significant differences in the maximum standardized uptake (SUVmax) value between these 2 types of pancreatic malignancies were found, and semiquantitative analysis using SUVmax cannot be used as a criterion for differentiation. However, pancreatic surgeons should consider the PET/CT scan as a necessary choice when the patient has a previous history of cancer at other organs.^[9]^ Except for the imaging findings, surgeons can also distinguish metastatic disease to the pancreas from primary pancreatic cancer by multiple serum parameters. Primary pancreatic cancer was always associated with elevated CA199, total bilirubin, and fasting plasma glucose levels.^[7]^ Compared to CECT and PET/CT, EUS has an inherent advantage of being able to obtain pathological proof by performing FNA and immunocytochemistry. Sox-10 staining has been demonstrated to be useful in identifying metastatic pancreatic malignancy and establishing a definitive pathological diagnosis.^[10]^ There are also a few slight differences between pancreatic metastases and primary pancreatic cancer. The former one is more likely to have well-demarcated tumor margins and appears hypoechoic, heterogeneous, lobular, and round when considering metastatic melanoma.^[11]^ So, EUS with FNA is recommended when facing inconclusive imaging studies of pancreatic lesions.

Melanoma can be categorized into cutaneous and extracutaneous melanomas (ECM) which is comprised of ocular (OM), mucosal (MM), and leptomeningeal melanomas. The incidence rate for both OM (5.6 per million person-years) and MM (2.3 per million person-years) are very low compared to CM (171.6 per million person-years). And, the 5-year relative survival rate of MM (34%) and OM (78.4%) are significantly lower than CM (89%).^[2]^ Although MM could be identical morphologically to its cutaneous counterpart, MMs have more aggressive biological manners and poorer outcomes than CM. MM are more likely to be detect at an advanced stage due to their hidden locations and a lack of symptoms. And, patients with MM are more susceptible to lymph nodal infiltration and distant visceral metastases. Moreover, at a molecular level, MM has been proven to be different from its cutaneous counterpart such as a higher rate of KIT aberrations (mutations or copy number increase) and a less frequency of BRAF mutations.^[12]^ These could be the explanations for the relatively poor survival outcomes of MM compared with OM. Moreover, a Denmark national study demonstrated that the independent predictor of MM of head and neck is R0 resection with age < 65, no distant metastases, and low TNM stage also being predictors of overall survival. The authors highlighted that R0 resection is the most important predictors of long-term survival and patients with negative margins have the lowest recurrence rate.^[13]^ The prognosis of metastatic melanoma is poor and each site is associated with different overall survival. Among all stage IV melanomas, the prognosis is best for metastases confined to skin tissue and lymph nodes, intermediate for metastases to lung and worst for all other organs, such as pancreas.^[14]^

Most patients with a metastatic pancreatic malignancy are usually not candidates for surgical treatment due to their widespread disease. The patient with metastases confined to parenchyma of pancreas at the time of diagnosis is a rare clinical case, accounting for 5% of all pancreatic neoplasms.^[3]^ According to a review article of 418 patients diagnosed with metastatic pancreatic diseases, the primary tumors were renal cell cancer (70.1%), melanoma (9.1%), colorectal cancer (8.6%), breast cancer (4.5%), sarcoma (4.3%), and lung cancer (3.4%).^[1]^ Many researchers have indicated that patients with metastatic pancreatic malignancy from renal cell cancer would have better overall survival with a 5-year survival rate of 63% compared to those with other primary cancers.^[1–4,15]^ The overall survival may differ from various pathological types. The 5-year survival rate for malignancies from sarcoma, breast cancer, and colorectal cancer is 32.4%, 34.3%, and 41.6%, respectively.^[3]^ The 1, 2, and 3-year survival rate are 44%, 33%, 22% for patients with pancreatic metastases from melanoma after radical surgery.^[2]^ Nevertheless, we believe that with advanced technology and annually decreasing morbidity and mortality rate in high-volume pancreatic centers, surgical treatment should be offered to patients with metastatic pancreatic malignancy from melanoma. Radical surgery with negative margins may be the only chance of cure for these patients. For metastases from renal cell cancer, the overall survival time is 52.6 months in surgical treatment patients and 11.2 months in nonoperative patients with a significance of 0.019, which is also a powerful proof for an aggressive surgical procedure.^[4]^ The important value of surgery as a prognostic predictor was also confirmed by multivariate analysis by Masetti et al,^[3]^ which indicated patients undergoing surgical treatment with negative margins had a statistically lower risk of earlier mortality. Other prognostic factors include the presence of clinical presentation and disease-free interval, which is confirmed in overall univariate survival analysis (*P* = .001 and *P* = .017, respectively).^[3]^

As for pancreatic metastases from melanoma, Deutsch et al^[2]^ argued that the overall survival is significantly superior in these patients undergoing a surgical procedure (18 months) compared to those receiving nonoperative protocol (7 months). The 1-, 2-, and 3-year survival rate are 44%, 33%, 22% for patients after radical surgery and 31%, 19%, and 15% for nonoperative patients. Among all these types of abdominal metastases from melanoma, the finest overall survival outcomes were detected in patients with GI metastases after surgery. The 1-, 2-, and 3-year survival rate is 52%, 41%, 32%, respectively, which is also better than those of nonoperative patients. Survival benefits were also detected in patients with other abdominal sites malignancy receiving metastasectomy versus those treated nonsurgically. And according to a retrospective study involving 54 patients, short-term outcomes related to surgical treatment are also favorable. No 30-day mortality was observed, and the complication rate was 11.1% (wound infection, n = 5; anastomotic leak, n = 1). And, 33 out of 36 patients who were symptomatic preoperatively received objective symptom relief. Generally, surgical treatment is practicable and associated with significantly improved overall survival at all abdominal sites.^[2,14]^

Complete removal with negative margins is the basic treatment modality for resectable metastases. There are multiple nonoperative therapies following surgery or treating patients with unresectable metastatic lesions. Melanoma is considered as a chemotherapy-resistant malignancy resulting in a low response rate in treating metastatic lesions. Chemotherapy with dacarbazine results in median survival time of only 7 to 9 months and, so far, no other chemotherapeutic agents or strategies have shown superiority to dacarbazine regarding long-term survival.^[16]^ In the modern era of evolving targeted therapies and immunotherapies, metastases from melanoma is the first solid malignancy to benefit from these revolutions and becomes the focus point in this novel therapeutic area.^[17]^ New therapeutic protocols have been proved to improve long-term survival and already used in routine clinical practice. The greatest survival improvement has been detected in the combination of BRAF with MEK inhibitor and PD-1 blocker. The survival proportion of patients at 12 months is 71.9% for PD-1 blocker and 74.5% for combination of BRAF with MEK inhibitor, respectively. The long-term outcomes are quite similar between single PD-1 blocker and combination with ipilimumab. No other treatment strategies for unresectable metastatic melanoma have been proved to be superior to these 2 protocols in terms of survival. Ugurel et al^[16]^ noted that the worst survival is detected with single ipilimumab or any chemotherapeutic agent, which is also a confirmation of our conclusion. It should be noted that the long-term follow-up information was only available for ipilimumab, which indicates a survival curve plateau around year 3.

Twenty-two percent of all unresectable or metastatic melanoma surviving at year 3 are alive at year 5 and beyond.^[18]^ Although not enough long-term follow-up data are available, this also indicates the longevity of benefit of the combination of BRAF with MEK inhibitor and PD-1 blocker.

In the last few years, the IHC markers have been widely used in the clinical setting in the diagnosis of pigmented lesions, such as S-100 and SOX-10. S-100 is the first IHC marker proved to be useful in the diagnosis of melanoma. This marker is associated with very high sensitivity (93–100%) but relatively low specificity.^[19]^ S-100 can be detected in all subtypes of melanoma and very useful in distinguishing melanocytic from nonmelanocytic tumors. Recently, SOX-10 has evolved as a novel IHC marker with high sensitivity and specificity. Except for melanoma, this IHC marker can be expressed in just 12% of all breast carcinomas, and no SOX-10 can be detected in the carcinoma of lung, colon, endometrium, and ovary. Vrotsos et al also demonstrated that SOX-10 is more sensitive and specific than S-100 and KBA62 in identifying metastatic melanoma in lymph nodes. So, SOX-10 is useful in the detection of micrometastases in sentinel lymph nodes. However, these 2 IHC markers cannot differentiate between benign and malignant pigmented skin lesions.^[20]^ So, S-100 and SOX-10 should be used combined with other IHC markers. Differentiation between malignant melanoma and a benign melanocytic lesion is crucial for identifying melanomas and subsequently improving the patients’ long-term survival. Chin et al^[21]^ indicated that malignant melanomas present with significant staining for phosphorylated CSE1L (100%) and only faint staining for the benign nevi (0%). Lyu et al noted that the number of p-Akt-positive cells in benign nevi is smaller than that of melanoma. The expression of p-Akt would be increased in melanoma with decreasing PTEN level, particularly in advanced cases.^[22]^ Although not enough relevant data are available, these novel IHC markers may aid in the differential diagnosis of malignant melanomas from benign pigment lesions.

## 4 Conclusion

Pancreatic metastases from other malignant tumors are a sporadic clinical condition and account for approximately 2% of all pancreatic malignancies. Metastatic pancreatic tumors are often associated with well-defined margins, tumor necrosis, enhancement, and distant metastases without pancreatic duct dilatation and parenchymal atrophy. As the most common type of metastatic pancreatic tumor, renal cell cancers tend to have higher attenuation values than that of primary pancreatic cancer, while they had similar attenuation values on portal phase. Despite CECT, PET/CT should be considered seriously when the patient has a previous history of cancer at other organs. Except for the imaging findings, surgeons can also distinguish metastatic disease to the pancreas from primary pancreatic cancer by multiple serum parameters. Primary pancreatic cancer was always associated with elevated CA199, total bilirubin, and fasting plasma glucose levels. Surgical resection for metastases to pancreas should be aggressively considered in selected patients due to its unique value of providing palliation and a chance to cure. For patients with unresectable lesions, in the modern era of evolving targeted therapies and immunotherapies, new therapeutic protocols should be recommended such as the combination of BRAF with MEK inhibitor and PD-1 blocker with or without ipilimumab.

## Author contributions

**Investigation:** Jianzhang Qin.

**Resources:** Xueqing Liu, Xiangyan Yin, Guiqing Meng.

**Software:** Tianyang Wang.

**Validation:** Jianhua Liu.

**Visualization:** Changqing Yan.

**Writing – original draft:** Feng Feng, Zhongqiang Xing, Jiayue Duan.

**Writing – review & editing:** Chen Liu, Jianhua Liu.

## References

[1] Sperti C Moletta L Patane G Metastatic tumors to the pancreas: the role of surgery. World J Gastrointest Oncol 2014;6:381–92. 25320654 10.4251/wjgo.v6.i10.381 PMC4197429

[2] Deutsch GB Flaherty DC Kirchoff DD Association of surgical treatment, systemic therapy, and survival in patients with abdominal visceral melanoma metastases, 1965–2014. JAMA Surg 2017;152:672. 28384791 10.1001/jamasurg.2017.0459 PMC5547921

[3] Masetti M Zanini N Martuzzi F Analysis of prognostic factors in metastatic tumors of the pancreas: a single-center experience and review of the literature. Pancreas 2010;39:135–43. 19820422 10.1097/MPA.0b013e3181bae9b3

[4] Schwarz L Regenet N Mabrut JY Long-term survival after pancreatic resection for renal cell carcinoma metastasis. Ann Surg Oncol 2014;21:4007–13. 24879589 10.1245/s10434-014-3821-4

[5] Sugimoto M Gotohda N Kato Y Pancreatic resection for metastatic melanoma originating from the nasal cavity: a case report and literature review. Anticancer Res 2013;33:567–73. 23393350

[6] Konstantinidis IT Dursun A Zheng H Metastatic tumors in the pancreas in the modern era. J Am Coll Surg 2010;211:749–53. 21109158 10.1016/j.jamcollsurg.2010.08.017 PMC3135384

[7] Yun HS Min YW Lee MJ Clinicoradiologic characteristics and outcomes of metastatic cancer to the pancreas and double primary pancreatic cancer. Clin Res Hepatol Gastroenterol 2013;37:182–8. 22749698 10.1016/j.clinre.2012.05.013

[8] Kang TW Kim SH Lee J Differentiation between pancreatic metastases from renal cell carcinoma and hypervascular neuroendocrine tumour: use of relative percentage washout value and its clinical implication. Eur J Radiol 2015;84:2089–96. 26318820 10.1016/j.ejrad.2015.08.007

[9] Hu S Zhang J Zuo C (18)F-FDG-PET/CT findings in pancreatic metastasis. Radiol Med 2015;120:887–98. 25795439 10.1007/s11547-014-0473-1

[10] Pang JC Roh MH Metastases to the pancreas encountered on endoscopic ultrasound-guided, fine-needle aspiration. Arch Pathol Lab Med 2015;139:1248–52. 26414469 10.5858/arpa.2015-0200-RA

[11] Jana T Caraway NP Irisawa A Multiple pancreatic metastases from malignant melanoma: conclusive diagnosis with endoscopic ultrasound-guided fine needle aspiration. Endosc Ultrasound 2015;4:145. 26020050 10.4103/2303-9027.156746 PMC4445173

[12] Bishop KD Olszewski AJ Epidemiology and survival outcomes of ocular and mucosal melanomas: a population-based analysis. Int J Cancer 2014;134:2961–71. 24272143 10.1002/ijc.28625

[13] Lawaetz M Birch-Johansen F Friis S Primary mucosal melanoma of the head and neck in Denmark, 1982–2012: demographic and clinical aspects. A retrospective DAHANCA study. Acta Oncol 2016;55:1001–8. 27031263 10.3109/0284186X.2016.1143117

[14] Reddy S Wolfgang CL The role of surgery in the management of isolated metastases to the pancreas. Lancet Oncol 2009;10:287–93. 19261257 10.1016/S1470-2045(09)70065-8

[15] Gutman H Hess KR Kokotsakis JA Surgery for abdominal metastases of cutaneous melanoma. World J Surg 2001;25:750–8. 11376411 10.1007/s00268-001-0027-2

[16] Ugurel S Röhmel J Ascierto PA Survival of patients with advanced metastatic melanoma: the impact of novel therapies. Eur J Cancer 2016;53:125–34. 26707829 10.1016/j.ejca.2015.09.013

[17] Simon A Kourie HR Kerger J Is there still a role for cytotoxic chemotherapy after targeted therapy and immunotherapy in metastatic melanoma? A case report and literature review. Chin J Cancer 2017;36:10.1186/s40880-017-0179-6 PMC5237156 28086948

[18] Schadendorf D Hodi FS Robert C Pooled analysis of long-term survival data from phase II and phase III trials of ipilimumab in unresectable or metastatic melanoma. J Clin Oncol 2015;33:1889–94. 25667295 10.1200/JCO.2014.56.2736 PMC5089162

[19] Ordã Ã Ez NG Value of melanocytic-associated immunohistochemical markers in the diagnosis of malignant melanoma: a review and update. Hum Pathol 2014;45:191–205. 23648379 10.1016/j.humpath.2013.02.007

[20] Mohamed A Gonzalez RS Lawson D SOX10 expression in malignant melanoma, carcinoma, and normal tissues. Appl Immunohistochem Mol Morphol 2013;21:506–10. 23197006 10.1097/PAI.0b013e318279bc0a

[21] Chin SY Wu PR Shih YH High expression of cytoplasmic phosphorylated CSE1L in malignant melanoma but not in benign nevi: phosphorylated CSE1L for the discrimination between melanoma and benign nevi. Int J Clin Exp Pathol 2015;8:1393–401. 25973023 PMC4396273

[22] Lyu SM Wu JY Byun JY Expression of phosphatase and tensin homologue, phospho-Akt, and p53 in acral benign and malignant melanocytic neoplasms (benign nevi, dysplastic nevi, and acral melanomas). Ann Dermatol 2016;28:548. 27746632 10.5021/ad.2016.28.5.548 PMC5064182

